# Systemic stomatal immunity: the beacon of fire and smoke in plant disease resistance

**DOI:** 10.1007/s44307-026-00098-8

**Published:** 2026-02-12

**Authors:** Changzhen Liu, Qiangsheng Yu, Susheng Song, Tiancong Qi

**Affiliations:** 1 State Key Laboratory of Green Biomanufacturing, Tsinghua-Peking Center for Life Sciences, School of Life Sciences, Tsinghua University, Beijing, 100084 China; 2 College of Life Sciences, Capital Normal University, Beijing, 100048 China

## Introduction

Plant diseases have long been a significant factor hindering agricultural. Current research indicates that most pathogenic bacteria and some fungi, among other pathogenic microorganisms, exploit stomata as key entry points to invade plants and cause diseases, including many globally prevalent ones (Melotto et al. 2008). In response, plants have evolved stomatal immune pathways to prevent pathogen invasion, which plays an extremely vital role in protecting plants from pathogen damage. Traditional stomatal immunity refers to the rapid response of local leaves, which close their stomata to block pathogen entry upon invasion. This pathway relies on the classic receptor complex module FLAGELLIN SENSING2 (FLS2)/BRI1-ASSOCIATED RECEPTOR KINASE 1 (BAK1) and the hormone pathways salicylic acid and abscisic acid (Hou et al. 2024). Recently, Liu et al. discovered a novel layer of immune coordination, termed systemic stomatal immunity (SSIM), that enables plants to rapidly synchronize stomatal defense across spatially separated leaves (Liu et al. 2026).

## Discovery of SSIM

Classical stomatal immunity has been known for years (Melotto et al. 2006), yet whether uninfected leaves could rapidly respond by closing their stomata in advance had remained unclear. Liu et al. stimulated local leaves of *Arabidopsis* with pathogens or pathogen-associated molecular patterns, and observed the stomatal aperture of systemic leaves (Liu et al. 2026). These results demonstrated that when local leaves perceived pathogen signals, the stomata of systemic leaves were induced to close. Liu et al. termed this newly discovered guard cell-based systemic defense system as “systemic stomatal immunity” (Liu et al. 2026). Distinct from classical systemic acquired resistance (SAR), which emerges over days and relies largely on salicylic acid-dependent transcriptional reprogramming (Fu and Dong 2013), SSIM came up on a markedly accelerated timescale. Local pathogen perception triggered stomatal closure in distal, untreated leaves within approximately 2.5 h. Further analyses revealed that this response also occurred in canonical SAR-defective mutant background, underscoring SSIM as a mechanistically independent yet complementary strategy of systemic immunity.

## SSIM is mediated by a mobile peptide encoded by an uORF

Liu et al. hypothesized the existence of a signaling molecule that could mediate SSIM (Liu et al. 2026). By integrating transcriptome and ribosome profiling, Liu et al. identified an immune-inducible upstream open reading frame (uORF) embedded within the *MYB51* locus. This *uORF*^*MYB51*^ encodes a 22-amino acid micropeptide, designated USIC ((uORF)-encoded Systemic Stomatal Immune Conductor). Upon immune activation, USIC accumulates in extracellular vesicles and can be transported from local leaves to systemic leaves through the phloem in the vascular tissue, as demonstrated by grafting assays and mass spectrometric detection. These findings position USIC as a bona fide long-distance signal that couples local immune perception to systemic physiological reprogramming (Liu et al. 2026).

## USIC mediates stomatal closure through a novel pathway

In systemic leaves, USIC is recognized by the plasma-membrane receptor complex formed by SUCROSE-INDUCED RECEPTOR KINASE 1 (SIRK1)-KINASE 7 (KIN7) (Liu et al. 2026). Ligand perception activates METACASPASE 4 (MC4) to proteolytically cleave KIN7. The intracellular domain of KIN7 then translocates from the plasma membrane to the tonoplast and promoted stomatal closure by interacting with *Arabidopsis* H ^+^ -ATPase 1 (AHA1) and PLASMA MEMBRANE INTRINSIC PROTEIN 2;1 (PIP2;1). Through this *FLS2*/salicylic acid/abscisic acid-independent cascade, an extracellular peptide signal is translated into coordinated intracellular organelle activity and rapid stomatal closure (Liu et al. 2026).

## Conceptual advances in immune signaling

Several conceptual advances emerge from this work (Liu et al. 2026). First, SSIM defines a fast-acting systemic immune response that complements the relatively slower but durable SAR pathway, revealing temporal stratification within plant immune networks. Second, the discovery of USIC highlights uORF-encoded micropeptide as an underappreciated reservoir of signaling molecules within organism-wide reach. Third, receptor cleavage-dependent signaling followed by subcellular relocalization provides a mechanistic framework linking surface immune perception to intracellular membrane and organelle dynamics.

## Application prospects of USIC

Beyond its mechanistic insights, the study raises intriguing translational possibilities (Liu et al. 2026). USIC is highly conserved in *Brassicaceae* plants and can stably exert its function in multiple *Brassicaceae* species, including oilseed rape (*Brassica napus*), Chinese cabbage (*Brassica rapa*), and radish (*Raphanus sativus*). Meanwhile, it can also promote stomatal closure in solanaceous plants such as tomatoes and *Nicotiana benthamiana*, demonstrating excellent application prospects. In the future, USIC may potentially be utilized as a plant-derived molecular regulator in agricultural production. Additionally, by using USIC as a template, applicable peptides might be discovered through artificial intelligence combined with machine learning approaches, enabling their application in important crops (Xiao et al. 2023; Wang and Xiao 2025).

## Conclusion

The stomatal immunity concept has been established for twenty years (Melotto et al. 2006), and Liu et al. discovered a novel plant immune pathway, SSIM, and provided a detailed analysis of its underlying molecular regulatory mechanisms (Liu et al. 2026), marking a milestone in the field of plant immunity research and a memory of pioneering scientists’ works. As a rapid systemic response mechanism, SSIM can effectively prevent plant diseases caused by pathogen invasion. The discovery of this process not only enhances researchers’ understanding of plant immunity but also offers new reference targets for agricultural production. Future efforts aimed at uncovering additional mobile micropeptides and integrating SSIM with established immune pathways promise to further illuminate how plants achieve whole-organism resilience in a pathogen-rich environment.
